# Preoperative incidence and risk factors of deep venous thrombosis in patients with isolated femoral shaft fracture

**DOI:** 10.1186/s12893-022-01534-x

**Published:** 2022-03-04

**Authors:** Weijie Yang, Qun Wei, Haicheng Wang, Kai Ding, Ming Li, Chao Li, Chunhui Liang, Yanbin Zhu, Wei Chen

**Affiliations:** 1 Department of Orthopaedic Surgery, The Third Hospital of Hebei Medical University; Orthopaedic Institution of Hebei Province; Key Laboratory of Biomechanics of Hebei Province, No. 139 Ziqiang Road, Shijiazhuang, 050051 People’s Republic of China; 2 Department of Hospital Infection Control, Department of Public Health, Hebei General Hospital, Shijiazhuang, 050051 Hebei People’s Republic of China; 3 Department of Pharmacy, The Third Hospital of Hebei Medical University, No. 139 Ziqiang Road, Qiaoxi District, Shijiazhuang, 050051 Hebei People’s Republic of China

**Keywords:** DVT, Femoral shaft fracture, Incidence, Risk factor

## Abstract

**Background:**

Preoperative deep vein thrombosis (DVT) of the lower extremities delays surgery in patients with femoral shaft fractures and impairs functional recovery. However, studies on preoperative DVT in patients with femoral shaft fractures are still rare. This study was aimed to retrospectively analyze the preoperative incidence, location and risk factors associated with DVT in patients with femoral shaft fractures.

**Methods:**

Data of patients with femoral shaft fractures and treated with surgery at the Third Hospital of Hebei Medical University were retrospectively collected from January 2013 to December 2019. The information collected included demographic data, comorbidities, injury-related data and laboratory tests. Patients were divided into DVT and non-DVT groups. Univariate and multivariate logistic regression analyses were performed to determine independent risk factors.

**Results:**

A total of 432 patients were included in this study, of whom 114 (26.4%) patients were diagnosed with preoperative DVT (all asymptomatic) and injured extremities of 78.1% (89/114) were investigated. Multivariate analysis showed that older age (increase in each 10 years), delay time from injury to operation (in each day), FIB > 4 g/L were independent risk factors for preoperative DVT.

**Conclusion:**

Patients with femoral shaft fractures (especially the elderly and patients with the above-mentioned conditions) are at the risk of DVT right from admission to surgery hence should be intensively monitored and provided with prompt treatment to prevent DVT.

## Introduction

Femoral shaft fractures account for 3.5% of all fractures in adults [1]. This type of fractures have a population-based incidence rate of between 19 and 21 per 100,000 person every years [2, 3]. Femoral shaft fractures, accompanied by soft tissue injuries that require surgical treatment, usually occur during high-energy trauma, such as traffic injuries [4]. However, emergency anesthesia and surgery often results in secondary trauma such as bleeding, requiring a delay in surgery [5]. During the waiting period for surgery, due to the fixation of the injured extremity, the lower extremity blood circulation can slow down. On the other hand, after trauma, the circulatory system is in a state of high coagulation and is hence prone to deep vein thrombosis (DVT) [6].

The use of rivaroxaban and low molecular heparin have now led to a marked improvement in the incidence of DVT [7]. Even so, the incidence of perioperative DVT in trauma patients is still as high as 5%–58% [8, 9]. Moreover, because asymptomatic DVT is easily overlooked by clinicians, pulmonary embolism resulting from thrombus extending proximally is fatal, with studies showing a global incidence of fatal pulmonary embolism of 0.66% to 7.50% [11].

Early detection and effective treatment of DVT can help reduce the incidence of death and adverse events. Previous studies of preoperative DVT in the orthopedic trauma field have mainly focused on hip fractures, tibial plateau fractures and calcaneal fractures and the reported incidence rate varies greatly, from 2.6 to 13% [12–14]. Moreover, the risk factors identified in the previous studies are not consistent and even contradictory [15]. Accordingly, it is possible that the findings available from these studies are not applicable or inappropriate for femoral shaft fractures.

To the best of our knowledge, there is no data regarding the preoperative incidence and risk factors of DVT in patients with femoral shaft fractures. This study aimed to retrospectively collect data of patients with femoral shaft fractures and investigate the incidence, location and risk factors associated with preoperative DVT.

## Methods

### Ethical considerations

This study was approved by the Ethics Committee of the Third Hospital of Hebei Medical University and the requirement for informed consent of all subjects was waived due to the retrospective design and the anonymized data. The study sample included patients with femoral shaft fractures admitted to the Third Hospital of Hebei Medical University between January 2013 and December 2019. The data of patients with femoral shaft fractures who received surgical treatment were collected.

### Inclusion and exclusion criteria

Patients aged 18 years and above, with an admission diagnosis of an isolated femoral shaft fracture that was finally surgically treated and with no anticoagulant use for 3 months before admission were included. The exclusion criteria were multiple fractures; open fractures; prior fractures (> 3 weeks from injury); pathological fractures; combined severe head, chest or abdominal trauma; fracture non-union; dyskinesia; history of autoimmune disease and thrombosis as well as incomplete medical records.

### Patient data and variables

The data covered four main areas: demographic data, comorbidities, injury-related data, and laboratory tests. General information collected was: sex, age, height, weight and calculated BMI as well as smoking and alcohol consumption history. The patients’ comorbidities included histories of cerebrovascular disease, lung disease, kidney disease, heart disease, hypertension, diabetes, surgery at any site for any reason; the American Society of Anesthesiologist (ASA) score. Data related to injury included injury mechanism (low or high-energy trauma), fracture type based on AO/OTA classification system, the time from injury to admission, the time from injury to DUS scanning and to operation as well as total hospital stay.

Laboratory examination indicators: blood samples were drawn from the patient's median cubital vein before any treatment after admission. Laboratory tests were performed to include the test indicators in this study. The indicators included the levels of total protein (TP), albumin (ALB), alanine transaminase (ALT), aspartate aminotransferase (AST), total bilirubin (TBIL), cholinesterase (CHE), high C-reactive protein (HCRP), lactic dehydrogenases (LDH), total cholesterol (TC), triglycerides (TG), glucose (GLU); counts of the following blood-cell types: white blood cells (WBC), neutrophiles (NEU), lymphocytes (LYM), red blood cell (RBC), platelets (PLT); hemoglobin (HGB), hematocrit (HCT), fibrinogen (FIB), fibrinogen degradation products (FDP) and D-dimer level.

### Management protocol of DVT

According to the institutional policy, after admission and blood drawing, all patients were treated with low-molecular weight heparin (3800 IU, subcutaneous injection, once per day) to prevent DVT (except patients with contraindications) and elevate of the injured lower extremity. All patients underwent routine Doppler ultrasound (DUS) bilateral lower extremity DVT tests 48 h after admission. The participants then underwent DUS tests every 72 h or when any symptoms suggestive of DVT was observed until surgery was carried out. The “Guidelines for diagnosis and treatment of deep vein thrombosis (2016 3rd Edition)” issued by the Chinese Medical Association were followed for diagnosis and treatment of DVT [14]. Positive diagnostic criteria for DVT included loss or incompressibility of the vein, lumen obstruction or filling defects, lack of respiratory variability in the vein segments above the knee and insufficient flow of blood during compression of the leg and foot. According to the thrombotic test criteria, the ultrasound physician examined femoral vein trunk, femoral deep, superficial, popliteal, tibial and fibular veins of both lower extremities and reported the findings. Thrombi located in the femoral vein trunk, superficial femoral vein, deep femoral vein and popliteal vein, each or combined, were considered proximal DVT. On the other hand, thrombi in the tibial vein and fibular vein are defined as distal DVTs and mixed DVTs (both proximal DVTs and distal DVTs). According to DUS test results, patients with DVT were treated with anticoagulant therapy (enoxaparin, 20–40 mg or dalteparin, 2500–5000 IU, twice daily). For all the patients, timing of surgery was determined by a senior orthopaedic surgeon based on the patient's examination results and physical condition. Thrombi located in the small saphenous vein or great saphenous vein were excluded from this study because of their low clinical significance [16]. All the included patients were divided into DVT and non-DVT groups according to DUS results.

### Statistical methods

SPSS 25.0 software (IBM, Armonk, New York, USA) was used for statistical analysis. The measurement data were first determined using the Shapiro–Wilk test to determine their distribution status, normal or non-normal. Normal distribution data were expressed by mean ± standard deviation (SD) and an independent sample *t*-test was used to compare the differences between the two groups. The Mann–Whitney *U* test was used for non-normally distributed data. Categorical variables were evaluated using the Chi-square or Fisher's exact tests. Multivariate logistic regression was performed to analyze the P values < 0.10 in the univariate analyses. Statistically significant difference for all the analyses were set at P values < 0.05. Hosmer–Lemeshow (H–L) test was used to evaluate the fitting degree of the final model and P values > 0.05 represented the acceptable result.

## Results

During the study window period, it was found that there were 618 patients with femoral shaft fractures. A total of 186 patients were excluded from the study based on inclusion and exclusion criteria. At last, a total of 432 patients with femoral shaft fractures, including 295 males and 137 females were involved in this study. The average age of the included patients was 44.8 years (SD, 17.8 years; range, 18–90 years and median, 43.0 years) whereby 85.2% of patients were aged between 18 and 65 years. The average body mass index (BMI) of the patients was 23.8 (SD 4.1; range 14.6–43.2). The fractures which caused by high-energy trauma were 335(77.5%) of. It was found that there were 315 (72.9%) fractures classified as types A, 82 (19.0%) types B and 35 (8.1%) types C. The mean time from injury to admission for all patients was 1.6 days (SD, 3.3; range 0–21 days) whereas the mean time from admission to operation for all patients was 7.7 days (SD, 6.5, range: 0–45 days). Further it was found that the mean total hospitalization stay was 23.6 days (SD, 23.2; range 4–341 days). Details for DVT and non-DVT groups are as presented in Table 1.

**Table 1:** Univariate analyses of risk factors associated with preoperative DVT following Femoral shaft fracture

Variables	Number (%) of DVT (n = 114)	Number (%) of non-DVT (n = 318)	*P*
Age (years)	49.4 ± 17.9	43.2 ± 17.4	0.001
Gender			0.504
Male	75 (65.8)	220 (69.2)	
Female	39 (34.2)	98 (30.8)	
BMI (kg/m^2^)			0.292
18.5–23.9	63 (55.3)	158 (49.7)	
< 18.5	3 (2.6)	10 (3.1)	
24.0–27.9	28 (24.6)	107 (33.6)	
≥ 28.0	20 (17.5)	43 (13.5)	
Alcohol consumption	12 (10.5)	36 (11.3)	0.817
Current smoking	13 (11.4)	43 (13.5)	0.563
Hypertension	33 (28.9)	68 (21.4)	0.102
Diabetes mellitus	16 (14)	29 (9.1)	0.140
The history of cerebrovascular disease	7 (6.1)	29 (9.1)	0.323
History of any surgery	31 (27.2)	79 (24.8)	0.621
Time from injury to operation	13.8 ± 8.5	7.7 ± 6.5	< 0.001
Total hospital stay (days)	26.8 ± 16.6	22.4 ± 25.2	0.040
ASA (III and above)	36 (31.6)	81 (25.5)	0.208
Mechanism (high-energy)	86 (75.4)	249 (78.3)	0.530
Fracture type (AO/OTA)			0.135
A	75 (65.8)	240 (75.5)	
B	27 (23.7)	55 (17.3)	
C	12 (10.5)	23 (7.2)	
TP (< 65 g/L)	97 (85.1)	262 (82.4)	0.510
ALB (< 35 g/L)	100 (87.7)	247 (77.7)	0.021
ALT (> upper limit)	35 (30.7)	95 (29.9)	0.869
AST (> upper limit)	45 (39.5)	106 (33.3)	0.238
TBIL (> 21 umol/L)	30 (26.3)	81 (25.5)	0.860
CHE (< 5 ku/L)	42 (36.8)	99 (31.1)	0.265
HCRP (> 8 mg/L)	94 (82.5)	238 (74.8)	0.098
LDH (> 250 U/L)	60 (52.6)	155 (48.7)	0.476
TC (> 5.2 mmol/L)	5 (4.4)	17 (5.3)	0.689
TG (> 1.7 mmol/L)	14 (12.3)	46 (14.5)	0.563
GLU (> 6.1 mmol/L)	62 (54.4)	150 (47.2)	0.186
WBC (> 9.5*10^9^/L)	57 (50.0)	151 (47.5)	0.645
NEU (> 6.3*10^9^/L)	70 (61.4)	200 (62.9)	0.778
LYM (< 1.1*10^9^/L)	49 (43)	134 (42.1)	0.876
RBC < lower limit	102 (89.5)	256 (80.5)	0.029
HGB < lower limit	102 (89.5)	244 (76.7)	0.003
HCT < lower limit	105 (92.1)	262 (82.4)	0.013
PLT (> 300*10^9^/L)	21 (18.4)	60 (18.9)	0.916
FIB (> 4 g/L)	46 (40.4)	76 (23.9)	0.001
FDP (> 5 ug/L)	48 (42.1)	91 (28.6)	0.008
D-dimer (> 0.5 mg/L)	86 (75.4)	179 (56.3)	< 0.001

ALT, Alanine transaminase, reference range: female, 7–40 U/L; male, 9–50 U/L; AST, aspartate transaminase, reference range: female, 13–35 U/L; male, 15–40 U/L; RBC, red blood cell, reference range: Female, 3.5–5.0*10^12^/L; male, 4.0–5.5*10^12^/L; HGB, hemoglobin, reference range: Female, 110–150 g/L; male, 120–160 g/L; HCT, hematocrit, reference range: Female, 35–45%; male, 40–50%

A total of 114 patients were found to have preoperative DVT (all asymptomatic), indicating an incidence of 26.4%. However, there was no pulmonary embolisms detected in this study. The average interval between injury and initial diagnosis of DVT was 6.4 days (median, 4 days), ranging from 0 to 30 days. The patients dragonized with preoperative DVT within 2 days were 35.1% (40/114), 71.9% (82/114) were diagnosed within 7 days whereas 86.8% (99/114) were diagnosed within 14 days. It was noted that the time from injury to operation was statistically significant in both DVT and non-DVT groups (13.8 ± 8.5 vs 7.7 ± 6.5, P < 0.001). It was found that the patients with a preoperative DVT had a significantly longer total hospitalization stay compared with patients without DVTs (26.8 ± 16.6 vs 22.4 ± 25.2, P = 0.040).

Results of this study show that 93 (81.6%) patients had DVT in the injured extremity, 11 (9.6%) in the uninjured extremity and 10 (8.8%) patients had DVT in bilateral extremities (Table 2). Further, it was found that 14 (12.2%) patients developed proximal DVT, 50 (43.9%) had distal DVT and 50 (43.9%) had mixed DVT. It was noted that there was no thrombosis in the anterior tibial veins of the patients. However, a total of 256 clots were found in the other six veins, representing an average of 2.25 (range, 1–6) for each patient. This represented 13 clots in the femoral common vein, 21 in the superficial femoral vein, 4 in the deep femoral vein, 54 in popliteal vein, 72 in the posterior tibial vein and 92 clots in the peroneal vein.

**Table 2:** Distribution of DVT and femoral shaft fracture location in 114 patients with DVT

Femoral shaft fracture location (N)	DVT location (N/%)		
	Injured extremity	Non-injured extremity	Bilateral
Left fracture (57)	48 (42.1)	4 (3.5)	5 (4.4)
Right fracture (56)	45 (39.5)	7 (6.1)	4 (3.5)
Bilateral fracture (1)	–	–	1 (0.9)
Total (114)	93 (81.6)	11 (9.6)	10 (8.8)

The results of this study show that there were significant differences between DVT and non-DVT patients in terms of age, time from injury to operation, levels of ALB, RBC count, levels of HGB, levels of HCT, the levels of FIB and FDP and D-dimer (P < 0.05, Table 1). Further, the multivariate analyses showed that three risk factors were identified to be independently associated with DVT, age (increase in each 10 years) (OR = 1.20, P = 0.003), x the levels of FIB and FDP and D-dimer (OR = 1.46, P = 0.003) (Table 3). The results of H–L test demonstrated the good fitness of the final model (X^2^ = 8.797, P = 0.360; Nagelkerke R^2^ = 0.236).

**Table 3:** Multivariate analyses of risk factors associated with SSI following femoral shaft fracture

Variable	OR and 95%CI	*P*
Age (increase of every 10 years)	1.20 (1.10 to 1.30)	0.003
Time from injury to operation (in each day delay)	1.11 (1.07 to 1.15)	< 0.001
FIB (> 4 g/L)	1.33 (1.04 to 1.71)	0.003

OR, odd ratio; CI, confidence interval

## Discussion

This is the first retrospective cohort study to evaluate the preoperative incidence of DVT in patients with isolated femoral shaft fractures. The study focused on the incidence, location and risk factors associated with DVT after femoral shaft fractures. It was found that the preoperative incidence of DVT was 26.4% (114/432) and 78.8% (100/114) had distal and mixs DVT. The average time from injury to initial diagnosis of DVT was 3.9 days whereby 43 (37.7%) patients were diagnosed within 2 days after the fracture, 85 (74.6%) were diagnosed within 5 days and 96 (84.2%) were diagnosed within 7 days. It was evident that the increased age, delayed time from injury to operation and FIB > 4 g/L were independently correlated with preoperative DVT following isolated femoral shaft fracture.

In previous studies, the incidence of preoperative DVT in lower extremity fractures ranges from 16.3% to 52.5% [10, 17–19]. This could be caused by differences in study methods, fracture types, sample sizes and diagnostic indicators. In a study by Wang et al*.* [17] DUS was used to detect lower limbs of 129 patients with femoral shaft fracture and the incidence of preoperative DVT was 40.3%. Elsewhere, Zhang et al*.* prospectively studied 160 patients over 65 years of age with distal femoral fractures and found that the incidence of preoperative DVT detected by DUS was 52.5% (84/160) [18]. The results of the current study showed a low incidence of preoperative DVT (26.4%, 114/432).

The lower incidence of preoperative DVT observed in the present study may be due to the following reasons: First, the sample size of our study was larger than those of the previous studies (432 vs 129–160); Second, the average age of the included patients was 44.8 years old, which was lower than that in the study by Zhang et al. (58.8 years old). It was also found that DVT mainly occurred in the injured extremity and distal, with the incidence of 81.6% (93/114) and 87.8% (100/114), respectively. The results were in consonance with the findings of Wang et al. [17], who reported the incidence of 83.3% and 87.3% in the injured extremity and in the distal, respectively.

Age is an important factor in the formation of DVT, especially in patients with trauma [10, 18, 20]. Among the 432 patients (aged 18–90 years) with femoral shaft fractures in the present study, it was evident that each additional 10 years of age was associated with a 20% increase in risk of preoperative DVT (P < 0.002). In a previous study, Zhang et al.[10] analyzed the risk factors for perioperative DVT in 404 patients with lower extremity fractures and found that age was a risk factor for preoperative DVT (OR: 1.03; 95% CI: 1.01–1.04; P < 0.0001). According to Makhdom et al. [21] and Goel et al. [22] the age of patients over 40 years old was an important risk factor for DVT in lower extremity injuries. The findings of the two studies corroborates with those of the present study. That the increase in age, decreased elasticity of blood vessels and increases the risk of thrombosis, suggests that orthopedists should formulate reasonable treatment measures according to the age of patients with femoral shaft fractures to effectively prevent the occurrence of thrombosis.

It has been found that hypercoagulability occurs 24 h after trauma and can remain until 10–30 days after injury [23, 24]. This may be the physiological basis for DVT in fracture patients. Delayed injury to operation time for patients with fracture, is an important factor that contributes to the high incidence of preoperative DVT. It has been reported that the incidence of preoperative DVT was between 11.9 and 62% in patients with hip fracture who delayed surgery by 1 to 2 days [25–27]. In the current study, it was found that the incidence of DVT before femoral shaft fracture was 26.4%. The different incidence may be due to the different fracture sites, as mentioned early. A study by Smith et al. [27] found that the incidence of postoperative DVT in patients with lower limb fracture was 14.5 and 33.3% after 1 and 7 days, respectively. In the present study it was found that for each day delayed from injury to surgery, the risk of preoperative DVT increased by 12% and this confirmed that delayed operation is a risk factor for preoperative DVT. Therefore, operation time is the only controllable risk factor among the many risk factors. It is hence suggested that orthopedic surgeons should operate the patients as early as possible under the premise of safety. However, DVT should be timely and effectively prevented, diagnosed and treated.

It was also evident that, peripheral blood FIB > 4 g/L at admission were a risk factor for preoperative DVT. The FIB are related markers of coagulation and fibrinolysis system, which reflect the equilibrium state of coagulation and fibrinolysis in body. Several studies have shown that elevated levels of FIB is associated with high risk of DVT [28]. For instance, FIB has been reported as a major factor of blood viscosity and red blood cell aggregation [29]. Furthermore, this may also explain the reason why blood is hypercoagulable in the early stages of after trauma [24, 30]. According to Koster et al. [31] and Kamphuisen et al. [32] patients with FIB > 5 g/L have a fourfold increased risk of DVT compared with normal patients (FIB < 3 g/L). Therefore, that results of the current study show FIB > 4 g/L in 40.4% (46/114) of DVTs is consistent with the findings in the previous studies. That the patients with FIB > 4 g/L had a 46% increased risk of preoperative DVT suggests that special attention should be paid to patients with elevated FIB and high risk of DVT when reviewing the preoperative examination results of patients.

Although this study is the first to retrospectively analyze the incidence of preoperative DVT in femoral shaft fractures, it had some inevitable limitations. First, it was a retrospective study, which might have led to inaccuracy in data collection. Secondly, the data on blood transfusion, prescribed drug use for perioperative medical optimization or number of smoking cigarettes cannot be captured, which potential also had effect on DVT formation. Third, angiography is the gold standard for the diagnosis of thrombi. However, due to the invasive examination of intravenous angiography, this study used non-invasive Doppler ultrasound to diagnose the DVT. Hence there may be some missed cases, which might have underreported the incidence of DVT.

## Conclusion

In summary, this study found that the incidence of DVT after femoral shaft fracture is 26.4%. It was evident that most of the thrombosis occurred in the injured extremity and was more likely located in the distal extremity. Further, the independent risk factors for preoperative DVT were older age, delayed time to operation and FIB > 4 g/L. Therefore, it is suggested that orthopedists who manage patients with femoral fracture should emphasize on the possibility of blood clots and timely detection of thrombosis after admission. Further, to develop and implement effective thrombosis prevention and treatment emphasis should be put on reducing long waiting times for surgery and paying attention to changes in the blood clot index, particularly in elderly patients.

## Data Availability

Data produced and analysed in this study are available from the corresponding author on request. The confidential patient data should not be shared.
